# Fixed Drug Eruption: An Underrecognized Cutaneous Manifestation of a Drug Reaction in the Primary Care Setting

**DOI:** 10.7759/cureus.28299

**Published:** 2022-08-23

**Authors:** George Shaker, Teja Mehendale, Charles De La Rosa

**Affiliations:** 1 Dermatology, Nova Southeastern University Dr. Kiran C. Patel College of Osteopathic Medicine, Fort Lauderdale, USA; 2 Dermatology, Pontiac General Hospital, Pontiac, USA

**Keywords:** dermatology case report, fixed drug eruption, cutaneous adverse drug reaction, dermatological manifestaion, drug-reaction

## Abstract

Fixed drug eruptions (FDEs) are dermatological manifestations of drug reactions that often occur in the same location upon re-exposure to a drug. They usually appear as erythematous-violaceous, circular patches, but several different variants have been described. They can often present without any associated symptoms, but in some cases, patients may complain of pain and pruritus. The lesions are often underdiagnosed or mistaken for insect bites, urticaria, or erythema multiforme; thus, an effort to bring awareness to this condition is warranted.

We present a 33-year-old African-American female who presented to the urgent care presenting with several violaceous patches of varying sizes that started two days ago. The lesions were located on the left shoulder, abdomen, right flank region, and behind the right knee. The lesions were associated with mild pain and pruritus. She believed she was bit by insects but denied seeing any insects at home or participating in any recent outdoor activities. She stated that she had a similar rash in the exact locations five months ago. Upon survey of new medications, she stated that she recently started taking her medications again, which include: hydrochlorothiazide, semaglutide, vitamin D supplement, and ibuprofen. Examination of the skin revealed several black, non-blanching macules with a surrounding ring of erythema on the left shoulder (3 x 3cm), abdomen (4 x 3cm), right popliteal region (3 x 2cm), and right flank region (6 x 7 cm). She was prescribed a medium-dose topical steroid cream to apply to the skin twice a day to decrease the intensity of the inflammatory reaction and thus relieve her symptoms. She was also educated on FDEs and was advised to discontinue Ibuprofen, one of the most commonly implicated drugs in FDEs. Upon returning for a follow-up four weeks later, she noted that she discontinued Ibuprofen, and her cutaneous reactions had fully resolved.

This case illustrates the prompt and accurate diagnosis of FDE leading to discontinuation of the offending drug and resolution of symptoms. It also represents the essential questions to ask when suspecting FDE.

## Introduction

Fixed drug eruptions (FDEs) are cutaneous manifestations that are associated with the usage of certain drugs. There are over 100 drugs associated with FDEs, but some of the most commonly implicated drugs include trimethoprim-sulfamethoxazole (and other sulfonamides), naproxen, ibuprofen, tetracyclines, other antibiotics (ampicillin, metronidazole), and barbiturates. They are often underdiagnosed or mistaken for insect bites, urticaria, or other conditions; this is due to them being less common among drug reactions, multiple variants, and a general unfamiliarity of the condition by clinicians [1]. These cutaneous drug eruptions often appear as oval, erythematous patches but may have different clinical presentations based on the many variants of the condition. They may occur anywhere on the body, including the face, tongue, hands, feet, torso, extremities, and genitalia [2]. They tend to recur in the same location as previous reactions and, therefore, a history of previous lesions in a similar location should prompt the consideration of FDE. The lesions are benign but may cause distress secondary to pruritus, pain, and appearance. A detailed history and physical exam can lead to prompt recognition and discontinuation of the offending medication, which is ultimately the treatment for FDE.

## Case presentation

We present a 33-year-old African-American female with a past medical history of hypertension, diabetes, and dysmenorrhea who presents to the urgent care with complaints of painful and pruritic lesions that started two days ago. The lesions were located on the left upper arm, right upper abdomen, right flank, and the popliteal region of the right knee. The patient believed she was bit by insects but denied seeing any insects at home or participating in any recent outdoor activities. She stated that she had similar lesions in the exact locations five months ago, which she stated occurred around the same time she took Ibuprofen for menstrual cycle cramps. She denied any recent changes in soaps, laundry detergents, or lotions and any systemic symptoms. Upon survey of new medications, she stated that she recently started taking her medications again, which include: hydrochlorothiazide, semaglutide, vitamin D supplement, and Ibuprofen. She had never been vaccinated for COVID-19. 

**Figure 1:**
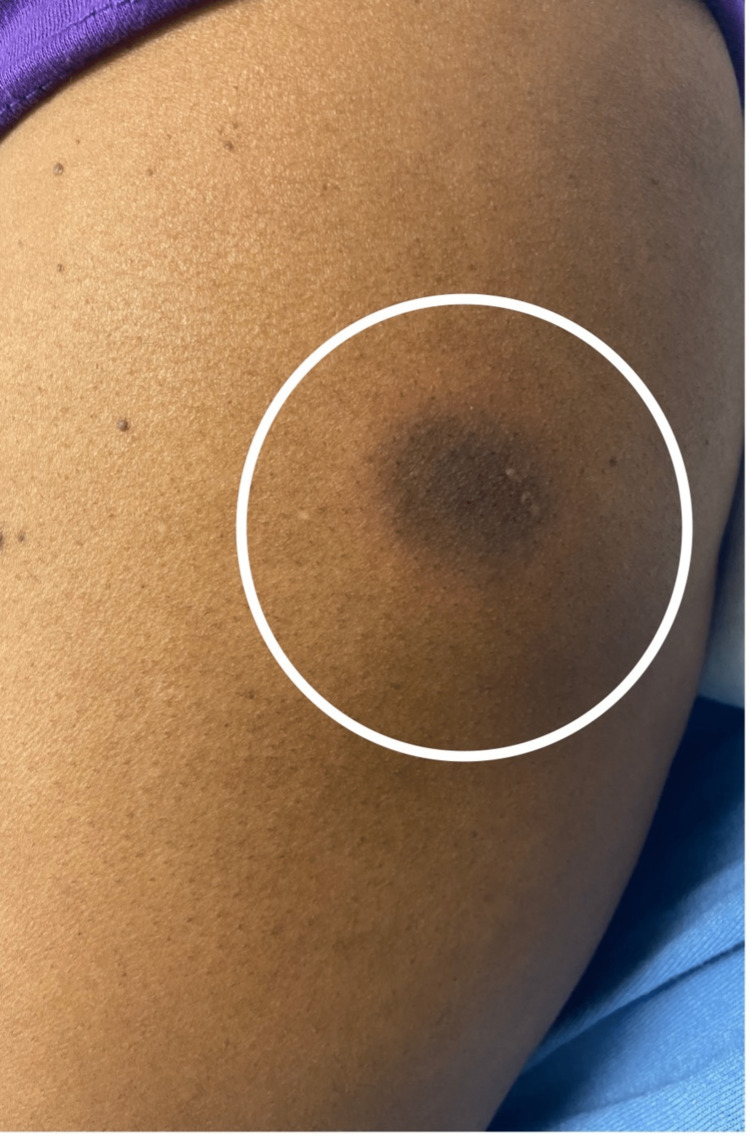
Violaceous well-demarcated patch with erythematous border on the left shoulder

**Figure 2:**
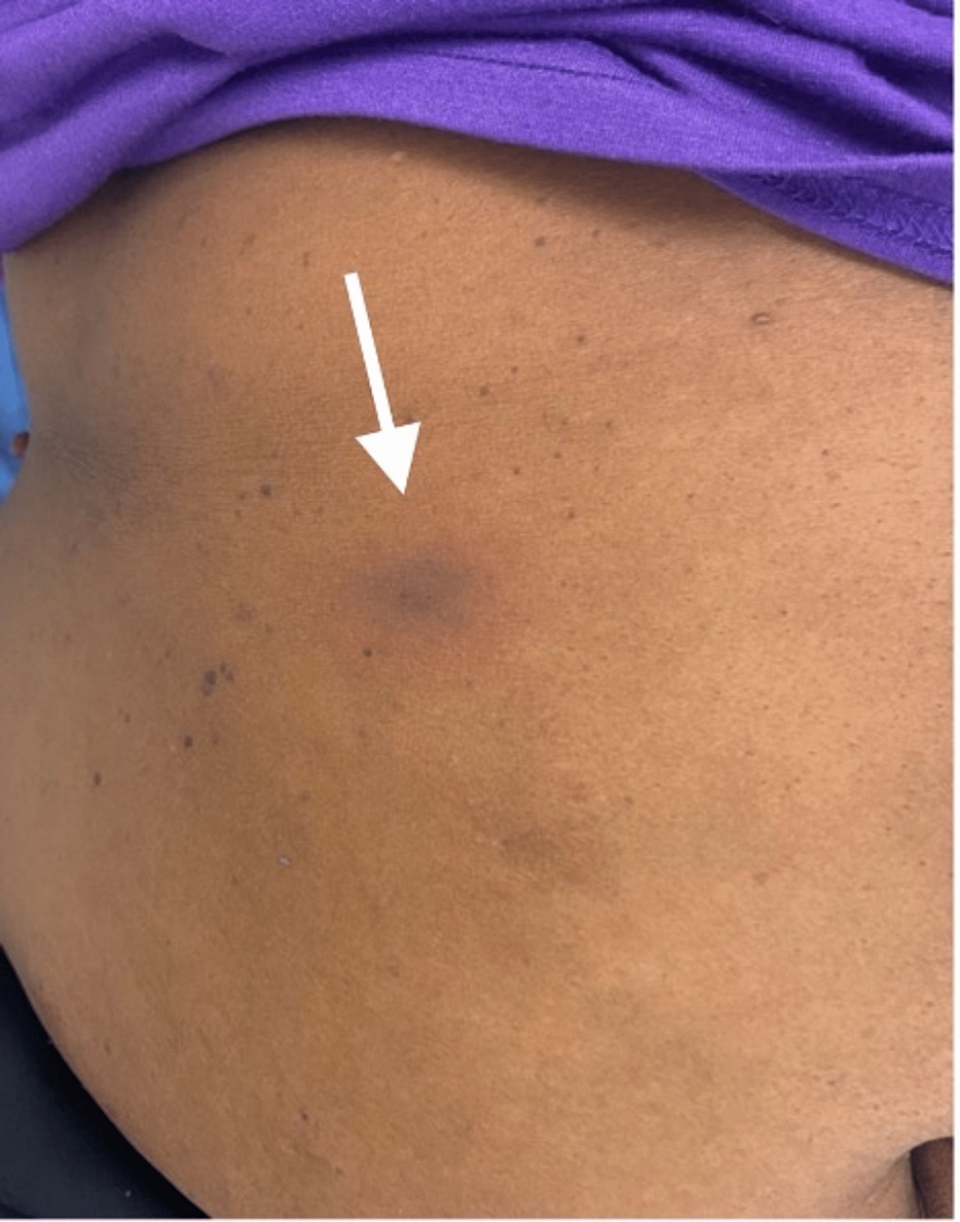
Violaceous poorly-demarcated patch with an erythematous border on the upper abdomen

**Figure 3:**
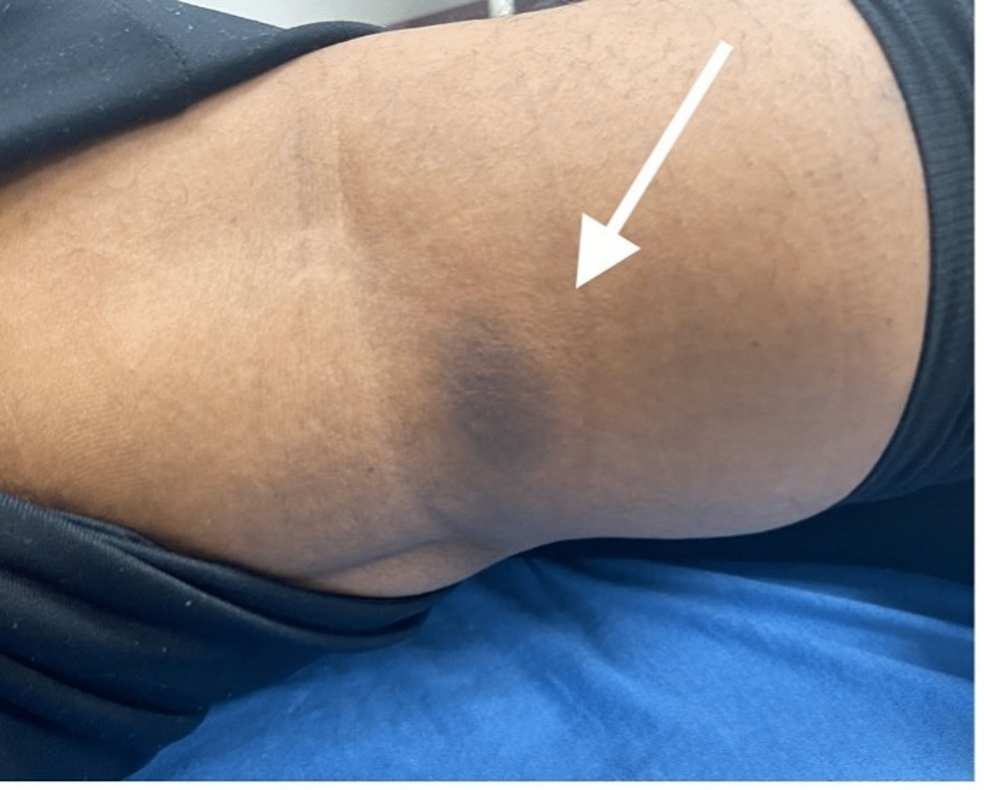
Violaceous well-demarcated patch with erythematous border on the right popliteal region

**Figure 4:**
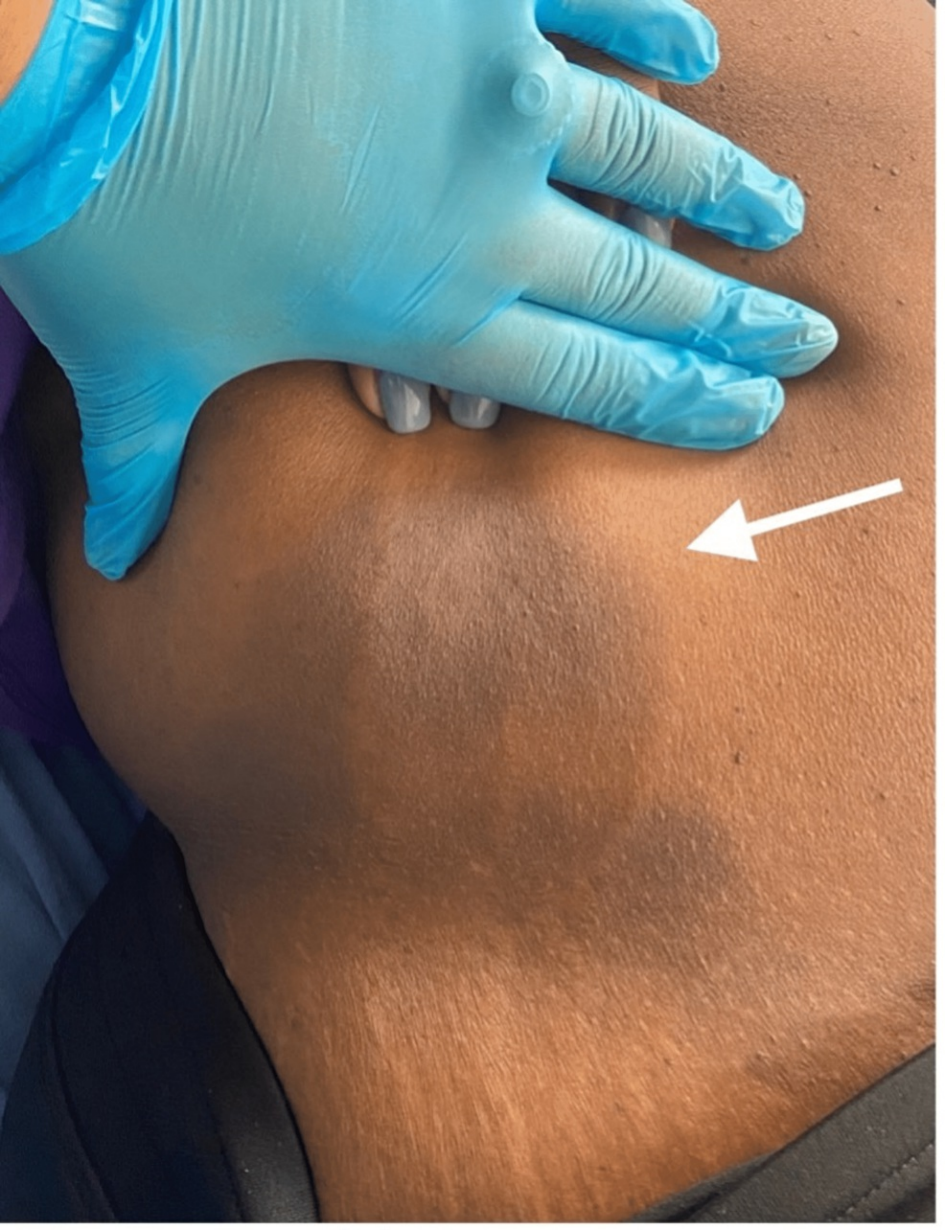
Violaceous poorly-demarcated patch with erythematous border on the right flank

Upon examination, the patient was afebrile and normotensive on presentation. Upon examination of the skin, several well-demarcated, non-blanching circular patches with a dusky violaceous appearance and surrounding rings of erythema were noted on the left arm, measuring 3 x 3cm (Figure 1), abdomen, measuring 4 x 3cm (Figure 2), right popliteal region, measuring 3 x 2cm (Figure 3), and right flank region, measuring 6 x 7 cm (Figure 4). No scaling, bleeding, or excoriation marks were present. 

She was prescribed a hydrocortisone ointment to apply to the skin twice a day to reduce the inflammatory reaction and thus diminish post-inflammatory pigmentation. She was also educated on FDEs and was advised to discontinue Ibuprofen. Ibuprofen was discontinued because the patient taking it for dysmenorrhea, a relatively benign condition, and Ibuprofen is one of the most commonly implicated medications in FDE. Upon returning for a follow-up four weeks later, she noted that she discontinued Ibuprofen, and her cutaneous reactions had fully resolved, with minimal pigmentation at the previous reaction locations. 

## Discussion

FDE is a cutaneous drug-related reaction that predominantly occurs in the same location(s) as previous reactions when exposed to an offending drug. FDEs are less common when compared to exanthematous skin reactions, which account for up to 95% of all skin reactions, and thus, are often underdiagnosed. In addition, there are several variants of FDEs, which makes diagnosing the reaction difficult if the clinician is unaware of variants of the condition.

These cutaneous lesions typically present as one or more well-demarcated, circular lesions with a dusky red or violaceous appearance [3]. The first reaction usually appears as a singular lesion, but with repeated exposure to an offending medication, a larger number of lesions can result [4]. Multiple variants, including linear, nonpigmenting, bullous, and generalized FDEs, along with many others, have been described. Systemic symptoms such as fever, chills, and fatigue are usually absent; however, the rash may be associated with pruritus, paresthesia, burning, and stinging sensations. Patients may attribute the lesion to what they believe was an insect bite, so the clinician must have a high degree of suspicion and take a detailed history to diagnose the FDE. Important questions to include in the history are if the patient had experienced previous lesions in the same area and if any new medications had been started recently. In addition, COVID-19 vaccination status should also be obtained as there have been cases of FDE after the Pfizer-BioNTech COVID-19 vaccination [5].

Although the pathophysiology behind the reaction is not completely understood, it is believed that the process is mediated by CD8(+) T cells. When the body is systemically exposed to an offending agent, that agent acts as a hapten that preferentially binds to basal keratinocytes, initiating an inflammatory response. CD8(+) T cells upregulate the production of cytokines, such as tumor necrosis factor-alpha and interferon-gamma, which perpetuate the inflammatory reaction. The initial eruption may take up to two weeks to manifest. Upon discontinuation of the offending agent, the hapten is no longer present and thus, the inflammatory response dissipates. The CD8(+) T cells give rise to memory T-cells which leads to a more rapid response upon the next exposure, usually occurring in the same location as the previous reaction and within minutes to hours of exposure [6]. 

There are many medications associated with FDEs. Some of the more common medications include antibacterial agents (trimethoprim-sulfamethoxazole, tetracyclines, penicillins, quinolones, dapsone), NSAIDs (acetylsalicylic acid, ibuprofen, naproxen, mefenamic acid), acetaminophen, barbiturates, antimalarials (quinine), and anticonvulsants (carbamazepine, lamotrigine) [7]. 

FDEs have no known racial or sex predilection, but one study concluded that there may be a genetic link between HLA-B22 to developing the reaction [8]. The prognosis is excellent, and the majority of patients generally make a full recovery upon discontinuation of the offending agent. Post-inflammatory hyperpigmentation is common after the resolution of the reaction and typically fades away after several months. It is important to educate patients on sun protection, such as covering areas with clothing and using sunscreen to expedite pigmentation resolution and prevent darkening [9]. 

In cases of uncertainty or for very rare variants of FDE, a biopsy can be obtained, which would show hydropic degeneration of the basal layer, dyskeratotic cells in the upper epidermis, and lymphocyte-rich dermal inflammatory infiltrate [10]. Other differential diagnoses include Stevens-Johnson syndrome/Toxic epidermal necrolysis, Bullous Pemphigoid, Psoriasis, Erythema Multiforme, Aphthous stomatitis, and autoimmune dermatitis. Management of FDEs should primarily consist of identifying and discontinuing the offending drug. Topical corticosteroids and antihistamines can provide symptomatic relief, although clinicians may be cautious with the use of levocetirizine and cetirizine as there have been cases of FDEs with the use of these antihistamines [11]. 

## Conclusions

FDEs are dermatological manifestations of a reaction due to certain medications. The diagnosis can typically be made through history and physical exam, although awareness of the variable presentations would likely assist in a prompt diagnosis. A history of a similar reaction in the same location in the setting of recently starting a new medication should clue the physician to the diagnosis. Patients often have post-inflammatory hyperpigmentation, which typically fades with time. FDEs are less common among drug reactions, have multiple variants, and are generally unfamiliar to clinicians in the primary care setting; this prompts the need to raise awareness of this condition. 
